# Comparative Analysis of Chloroplast *psbD* Promoters in Terrestrial Plants

**DOI:** 10.3389/fpls.2017.01186

**Published:** 2017-07-13

**Authors:** Shuichi Shimmura, Mikio Nozoe, Shota Kitora, Satoko Kin, Shigeru Matsutani, Yoko Ishizaki, Yoichi Nakahira, Takashi Shiina

**Affiliations:** ^1^Graduate School of Life and Environmental Sciences, Kyoto Prefectural University Kyoto, Japan; ^2^AMITA Institute for Sustainable Economies Co., Ltd. Kyoto, Japan; ^3^Kyoto Botanical Garden Kyoto, Japan; ^4^College of Agriculture, Ibaraki University Ibaraki, Japan

**Keywords:** *psbD* LRP, chloroplast, promoter, evolution, stress

## Abstract

The transcription of photosynthesis genes encoded by the plastid genome is mainly mediated by a prokaryotic-type RNA polymerase called plastid-encoded plastid RNA polymerase (PEP). Standard PEP-dependent promoters resemble bacterial sigma-70-type promoters containing the so-called -10 and -35 elements. On the other hand, an unusual light- and stress-responsive promoter (*psbD* LRP) that is regulated by a 19-bp AAG-box immediately upstream of the -35 element has been mapped upstream of the *psbD-psbC* operon in some angiosperms. However, the occurrence of the AAG-box containing *psbD* LRP in plant evolution remains elusive. We have mapped the *psbD* promoters in eleven embryophytes at different evolutionary stages from liverworts to angiosperms. The *psbD* promoters were mostly mapped around 500–900 bp upstream of the *psbD* translational start sites, indicating that the *psbD* mRNAs have unusually long 5′-UTR extensions in common. The -10 elements of the *psbD* promoter are well-conserved in all embryophytes, but not the -35 elements. We found that the AAG-box sequences are highly conserved in angiosperms and gymnosperms except for gnetaceae plants. Furthermore, partial AAG-box-like sequences have been identified in the *psbD* promoters of some basal embryophytes such as moss, hornwort, and lycophyte, whereas liverwort has the standard PEP promoter without the AAG-box. These results suggest that the AAG-box sequences of the *psbD* LRP may have evolved from a primitive type of AAG-box of basal embryophytes. On the other hand, monilophytes (ferns) use another type of *psbD* promoter composed of a distinct *cis*-element upstream of the potential -35 element. Furthermore, we found that *psbD* expression is not regulated by light in gymnosperms or basal angiosperms, although they have the well-conserved AAG-box sequences. Thus, it is unlikely that acquisition of the AAG-box containing *psbD* promoter is directly associated with light-induced transcription of the *psbD-psbC* operon. Light- and stress-induced transcription may have evolved independently and multiple times during terrestrial plant evolution.

## Introduction

Chloroplasts in plant and algal cells are semiautonomous organelles that have their own genome and gene expression system, reflecting their cyanobacterial origin. Chloroplast transcription is mediated by two distinct RNA polymerase systems, a prokaryotic multi-subunit RNA polymerase (PEP) whose core subunits are encoded by chloroplast genomes and single-subunit bacteriophage-type RNA polymerases (NEP) that are encoded by the nuclear genome (Hess and Börner, 1999; Liere et al., 2011; Yagi and Shiina, 2012, 2014; Liebers et al., 2017). The PEP core enzyme consists of four major subunits, designated as α, β, β′, and β″" subunits, which are homologous to bacterial subunits. Another dissociable subunit called a sigma factor allows the core enzyme to initiate transcription from the specific promoters. Multiple sigma factor genes have been identified in embryophytes (Tanaka et al., 1997; Morikawa et al., 1999; Fujiwara et al., 2000; Hara et al., 2001; Kasai et al., 2004; Kubota et al., 2007; Kanazawa et al., 2013), and they play specific roles in transcriptional regulation in response to developmental and/or environmental cues (reviewed by Kanamaru and Tanaka, 2004; Shiina et al., 2005; Schweer et al., 2010; Börner et al., 2015; Chi et al., 2015). Standard PEP-dependent promoters resemble bacterial sigma-70 type promoters containing -10 (TATAAT) and -35 (TTGACA) elements, reflecting their bacterial origin. NEP recognizes distinct types of promoters containing a core YRTA motif (Hess and Börner, 1999; Liere and Maliga, 1999; Liere et al., 2004; Börner et al., 2015). PEP transcribes mainly photosynthesis genes in mature chloroplasts while NEP transcribes housekeeping genes in both chloroplasts and non-photosynthetic plastids (Allison et al., 1996; Hajdukiewicz et al., 1997). The functional coordination of PEP and NEP plays a critical role in plastid differentiation in angiosperms.

In contrast to angiosperms, chloroplasts of the green algae *C. reinhardtii* harbor a simple transcription system, which is dependent on PEP and a single sigma factor SIG1 (Bohne et al., 2006; Yagi and Shiina, 2014). No NEP has been identified in *Chlamydomonas*. It is considered that embryophytes have developed complex transcription systems to adapt to marked environmental stresses. However, the evolutionary process of chloroplast transcription systems in embryophytes remains largely elusive.

Most PEP-dependent genes are actively transcribed in green tissues including the leaves. The chloroplast run-on experiments demonstrated that PEP-dependent transcription is activated by high light compared to normal growth light (Baena-González et al., 2001). However, the accumulation of most PEP-dependent transcripts is not regulated by light/dark transitions or environmental stresses, possibly due to the extraordinary stability of their transcripts (Shiina et al., 1998; Hayes et al., 1999). The only exception is a *psbD* light-responsive promoter designated *psbD* LRP, which is located upstream of a *psbD-psbC* operon encoding D2 (PsbD) and CP47 (PsbC) subunits of the PSII reaction center complex (Christopher et al., 1992; Wada et al., 1994; Allison and Maliga, 1995; To et al., 1996; Hoffer and Christopher, 1997). Transcription from the *psbD* LRP is activated by not only high-irradiance light, but also various abiotic stresses, including salt, high osmolarity and heat (Nagashima et al., 2004) and circadian rhythm (Noordally et al., 2013). The *psbD* LRP contains unique signature sequences named the AAG-box, immediately upstream of the -35 element (Allison and Maliga, 1995; Kim and Mullet, 1995; To et al., 1996; Nakahira et al., 1998; Kim et al., 1999). The AAG-box is composed of two different repeat units (AAGT and GACC/T repeats). *In vitro* transcription assays from the *psbD* LRP revealed that both repeat motifs are important for transcription, but not the -35 element in barley or wheat (Nakahira et al., 1998; Kim et al., 1999). Furthermore, the AAGT repeat interacts with the sequence-specific DNA-binding protein AGF (Kim and Mullet, 1995; Nakahira et al., 1998; Kim et al., 1999). It has also been shown that the stress-responsive plastid sigma factor SIG5 directs the activation of the *psbD* LRP in *Arabidopsis thaliana* (Nagashima et al., 2004; Tsunoyama et al., 2004; Onda et al., 2008).

The *psbD* promoter mapped in *Chlamydomonas* has a well-conserved -10 element, but lacks the AAG-box and standard -35 element (Klein et al., 1992; Klinkert et al., 2005). In addition, nucleotide sequence comparison of the upstream regions of the *psbD* among embryophytes suggests that *A. thaliana* (angiosperm) and *Pinus thunbergii* (gymnosperm) have the *psbD* LRP in their genome, but not the other basal embryophytes *Physcomitrella patens* (moss) and *Marchantia polymorpha* (liverwort) (Kanazawa et al., 2013). These findings suggest that the *psbD* LRP may have emerged during the evolution of embryophytes. However, evolution of the *psbD* promoter remains elusive. In this study, we mapped the promoter region of the *psbD-psbC* operon in eleven embryophytes at different evolutionary stages from liverwort to angiosperm. The results suggest that AAG-box sequences of the *psbD* LRP in angiosperms and gymnosperms may have evolved from the partial AAG-box-like sequences detected in the *psbD* promoters of basic embryophytes such as moss, hornwort, and lycophyte, while monilophytes (ferns) use a distinct type of *psbD* promoter lacking the AAG-box. On the other hand, light-dependent *psbD* expression was not observed in gymnosperms or primitive angiosperms that possess the well-conserved AAG-box, suggesting that the AAG-box containing *psbD* promoter acquisition is unlikely to be associated with the occurrence of light-dependent *psbD* expression.

## Materials and Methods

### Plant Materials and Growth Condition

For primer extension analysis, *A. thaliana*, *Adiantum capillus-veneris*, *P, patens*, and *M. polymorpha* (Tak-1) were grown in the light in growth chambers at 22°C under 16-h-light/8-h-dark (80–100 μmol photons m^-2^ s^-1^). Other samples (*Laurus nobilis*, *Ginkgo biloba*, *P. thunbergii*, *Equisetum hyemale*, *Psilotum nudum*, and *Lycopodium clavatum* were collected from plants cultivated at Kyoto Botanical Garden. Leaf samples were collected in the daytime, and immediately frozen in liquid N_2_. Light-induced gene expression analysis was carried out with plants (*A. capillus-veneris, C. revoluta, P. thunbergii, L. nobilis*, *A. thaliana*) grown in the growth chambers at 22°C under 16-h-light/8-h-dark (80 μmol photons m^-2^ s^-1^). Plants were dark-adapted for 72 h, and then exposed to light of 180 μmol photons m^-2^ s^-1^ for 4 h in the growth chamber. Collected leaf samples were immediately frozen in liquid N_2_.

Light-induced gene expression analysis (**Supplementary Figures S4**–**S8**) was also carried out with a wide range of embryophytes at different evolutionary stages from moss to angiosperms grown in the growth chambers at 22°C under 16-h-light/8-h-dark (80 μmol photons m^-2^ s^-1^). Plants were dark-adapted for 72 h (D), and then illuminated (275 μmolm^-2^s^-1^) for up to 12 h (L). Osmotic stress was achieved by 250 mM mannitol treatment for 6 h to the detached leaves. Collected leaf samples were immediately frozen in liquid N_2_.

*Arabidopsis thaliana* wild-type Columbia ecotype and *AtSIG5*-overexpressing plants were germinated and grown on two layers of filter paper on the one-half Murashige and Skoog (MS) medium containing 0.8% (w/v) agar at 22°C with 16-h light (80 μmol photons m^-2^ s^-1^)/8-h dark cycles for 10 days. For salt and high osmotic stress treatments, the seedlings were transferred to one-half MS medium containing 250 mM NaCl or 250 mM mannitol, and incubated under light (80 μmol photons m^-2^ s^-1^) for 6–24 h. For low temperature treatment, the seedlings were incubated at 4°C for 6–24 h under light conditions of 50 μmol photons m^-2^ s^-1^. For light response experiments, the seedlings were dark adapted for 72 h (D) and illuminated for 4 h with white light (80 μmol photons m–2 s–1) or a blue LED light (50 μmol photons m^-2^ s^-1^) or a red LED light (50 μmol photons m^-2^ s^-1^).

### Transgenic Plants

First strand cDNA of *AtSIG5* was synthesized from total RNA prepared from *Arabidopsis* seedlings using AMV reverse transcriptase (TaKaRa). cDNA was amplified by PCR using KOD-plus-DNA polymerase (TOYOBO) according to the manufacturer’s protocols. To obtain an AtSIG5 overexpression construct under the control of CaMV 35S promoter, the GUS gene of the binary vector pBI121 was replaced with the AtSIG5 cDNA. The resulting constructs were introduced into *Agrobacterium tumefaciens* and used to transform the wild-type (Col-0) plants.

### Total RNA Isolation, Primer Extension Analysis, and Northern Blot Analysis

Total RNA was extracted from the leaves using the RNeasy Plant Mini kit (Qiagen, United States) or TRIZOL^®^ following the manufacturer’s instructions. The primer extension assays were performed on the total RNA using the Primer Extension System (Promega) with the AMV reverse transcriptase following the manufacturer’s instructions. Primers used are listed in Supplementary Table S1. Primer extension products were analyzed on a 6% polyacrylamide-7 M urea sequencing gel. For northern blot analysis, total RNA samples (2 μg) were separated by denaturing agarose gelelectrophoresis. After capillary blotting onto Hybond-N nylon membrane, RNA gel blots were hybridized to the randomly primed DNA probes for *psbA* and *psbD* of each plant. The *psbD* UTR probe (-1085 to -726 of the *psbD* translation start codon) was designed to detect specifically the transcripts from the *psbD* LRP in *Arabidopsis*. The *psbD* and *psbA* coding region probes were designed to detect transcripts produced from all multiple promoters in the *psbD-psbC* operon. The *AtSIG5* probe was also prepared using specific primers. The psbD UTR probes specific for each plant were also generated by using PCR primers.

## Results

### *psbD* LRP Transcription Is Dependent on SIG5

**FIGURE 1:**
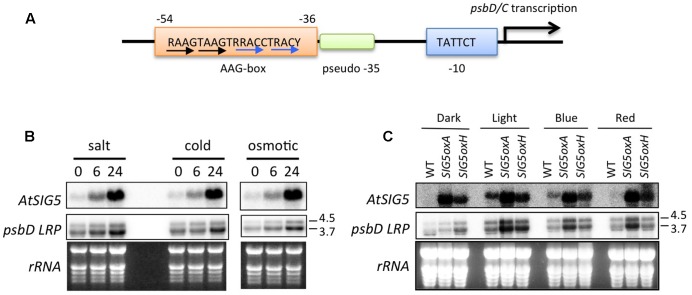
Involvement of AtSIG5 in transcription from the *Arabidopsis psbD* LRP. **(A)** Schematic structure of the *psbD* LRP in *Arabidopsis*. The *psbD* LRP consists of a well-conserved –10 element and an AAG-box upstream of a pseudo –35 element. The conserved 19-bp AAG-box that contains AAGT and GACC/T repeats (black and blue arrows, respectively) is indicated. R, A or G. Y, C or T. **(B)** Northern blot analysis of *psbD* LRP and *AtSIG5* transcripts in *A. thaliana* treated with salt (250 mM NaCl), cold (4°C), and osmotic (250 mM mannitol) stresses for indicated time periods. Total RNAs (2 μg) were electrophoresed in a denatured gel, blotted, and hybridized to ^32^P-labeled gene-specific probes, *psbD* LRP UTR and *AtSIG5* probes. The psbD probe was used to detect 3.7 and 4.5 kb mRNAs transcribed from the *psbD* LRP. Transcription of *psbD* LRP was significantly induced by abiotic stresses. **(C)** Light-dependent expression of *psbD* LRP transcripts in *AtSIG5* overexpressing plants. The seedlings were dark adapted for 72 h (D) and illuminated for 4 h with white light (80 μmol photons m^-2^ s^-1^) or a blue LED light (50 μmol photons m^-2^ s^-1^) or a red LED light (50 μmol photons m^-2^ s^-1^).

The *psbD* LRP is a unique PEP-dependent chloroplast promoter, which is responsible for the transcription of the *psbD-psbC* operon. The *psbD-psbC* operon is well-conserved among plants and cyanobacteria. Unlike standard PEP-dependent promoters composed of sigma-70 type -10 (TATAAT) and -35 (TTGACA) elements, it has been shown that *psbD* LRP activity is dependent on the upstream AAG-box in tobacco (Allison and Maliga, 1995), barley (Kim and Mullet, 1995), rice (To et al., 1996), and wheat (Nakahira et al., 1998; **Figure 1A**). On the other hand, the -35 element of the *psbD* LRP is not essential for transcription activity (To et al., 1996; Nakahira et al., 1998; Kim et al., 1999; Thum et al., 2001b). These findings suggest that the upstream AAG-box may take over the role of the pseudo -35 element in the *psbD* LRP.

Multiple promoters have been identified in the *psbD-psbC* operon (Hoffer and Christopher, 1997). The most upstream promoter (∼950 of the psbD translation start site) is a so-called *psbD* LRP that is specifically activated by blue light. To identify specifically mRNAs transcribed from the *psbD* LRP, we used a *psbD* UTR probe (-1085 to -726 of the *psbD* translation start codon) that is designed to be located upstream of the second promoter at -550 (Tsunoyama et al., 2004).

As shown in **Figure 1B**, the 4.5- and 3.7-kb transcripts from the *psbD* LRP were specifically detected by the *psbD* LRP UTR probe. As reported by Nagashima et al. (2004), various abiotic stresses including salt, cold, and hyperosmotic stresses induce transcription at the *psbD* LRP in a time-dependent manner (**Figure 1B**). Similarly, SIG5 transcription is activated by abiotic stresses. Previous reports (Nagashima et al., 2004; Tsunoyama et al., 2004) demonstrated that *psbD* LRP activity is abolished in *AtSIG5*-deficient mutants in *Arabidopsis*. In order to further define the role of AtSIG5 in transcription at the *psbD* LRP, we developed SIG5 overexpression lines (SIG5oxA and SIG5oxH) and examined transcription activity from the *psbD* LRP. The accumulation of *psbD* LRP transcripts was significantly increased by the overexpression of *AtSIG5* in illuminated plants irrespective of the presence of white, blue, and red light, but only slightly in the dark (**Figure 1C**). These results clearly demonstrate that SIG5 specifically mediates transcription from the *psbD* LRP in the light. Photoreceptors including CRY1, CRY2, and PhyA have been shown to be involved in the light-induced expression of the *psbD-psbC* operon in *Arabidopsis* (Thum et al., 2001a), while *AtSIG5* overexpression cannot activate transcription from the *psbD* LRP in the dark (**Figure 1C**). Taken together, photoreceptor-mediated signaling may modify SIG5 or SIG5 import in a light-dependent manner and activate transcription at the *psbD* LRP.

### *psbD* Transcripts have Markedly Long 5′-UTRs in Common

**FIGURE 2:**
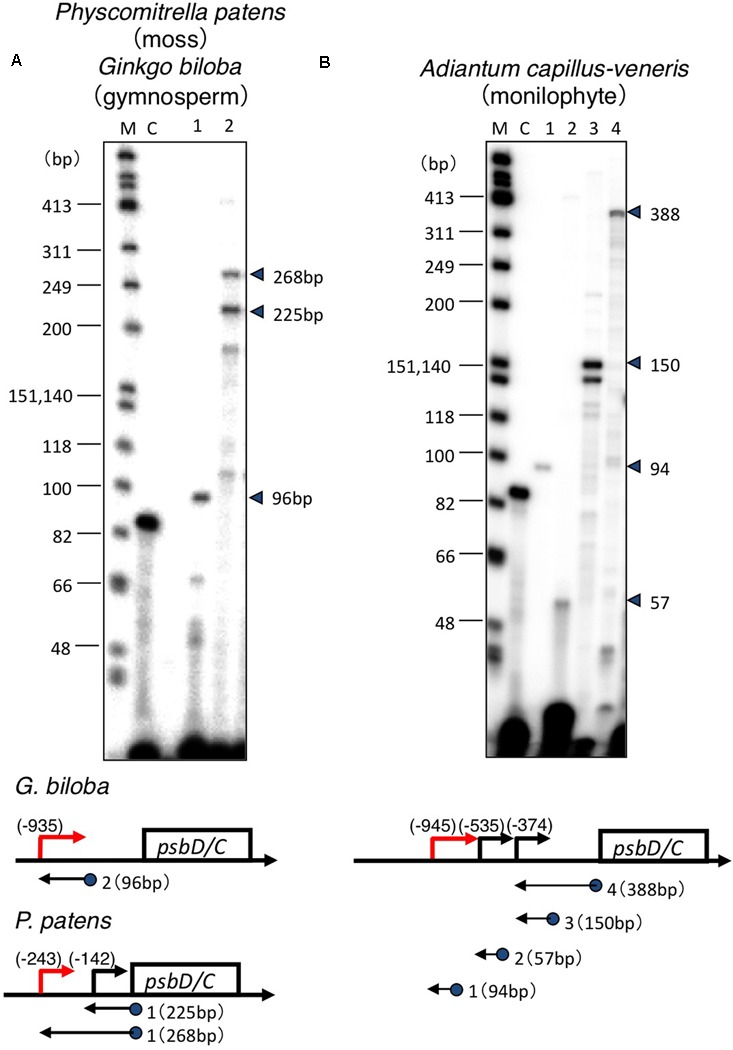
Mapping of the 5′ ends of the *psbD* transcripts. Analysis of the *psbD* transcripts of the moss *P. patens* and primitive gymnosperm *G. biloba* **(A)**, and monilophyte *A. capillus-veneris* **(B)** by primer extension assays. Primers used are indicated by numbers on the top of each lane. The size of the extension product is shown on the right. The position of primers and the size of the extension products are shown on the gene map. The deduced sites of the 5′-end of each transcript are shown as numbers in parentheses. Lane C shows an experiment with the control RNA and primer provided by the manufacturer that produces an 87-base primer extension product.

Transcription initiation sites of the *psbD-psbC* operon have only been identified in some angiosperm model plants, including barley, wheat, rice, tobacco, and *Arabidopsis* (Yao et al., 1989; Christopher et al., 1992; Wada et al., 1994; To et al., 1996; Hoffer and Christopher, 1997), and green algae *Chlamydomonas reinhardtii* (Klein et al., 1992). In order to address the evolutionary changes of *psbD* promoter structures, we mapped 5′-ends of *psbD* transcripts of eleven embryophytes at different evolutionary stages from liverworts to angiosperms using primer extension analysis (**Figure 2** and **Supplementary Figure S1**, **S2**). Leaf samples were collected from plants grown in Kyoto Botanical Garden in the daytime, except for *A. thaliana*, *A. capillus-veneris*, *P. patens*, and *M. polymorpha* that were grown in the light in growth chambers. We estimated the size of primer extension products approximately by comparing their mobility profiles to single-strand DNA ladders. In order to determine the start sites as exactly as possible, we designed appropriate primers that produce primer extension products shorter than 300 bases. Next we searched for sequences similar to the conserved -10 sequences (TATTCT) of the *psbD* LRP in close proximity to the identified transcription initiation sites. Then, we aligned the deduced *psbD* promoter sequences with those of other plants using the -10 element as reference. In this study, we considered the *psbD* transcripts with the most upstream terminus as the primary transcripts, except for *P. thunbergii* and *E. hyemale*, which have another promoter upstream of the potential *psbD* LRP.

**FIGURE 3:**
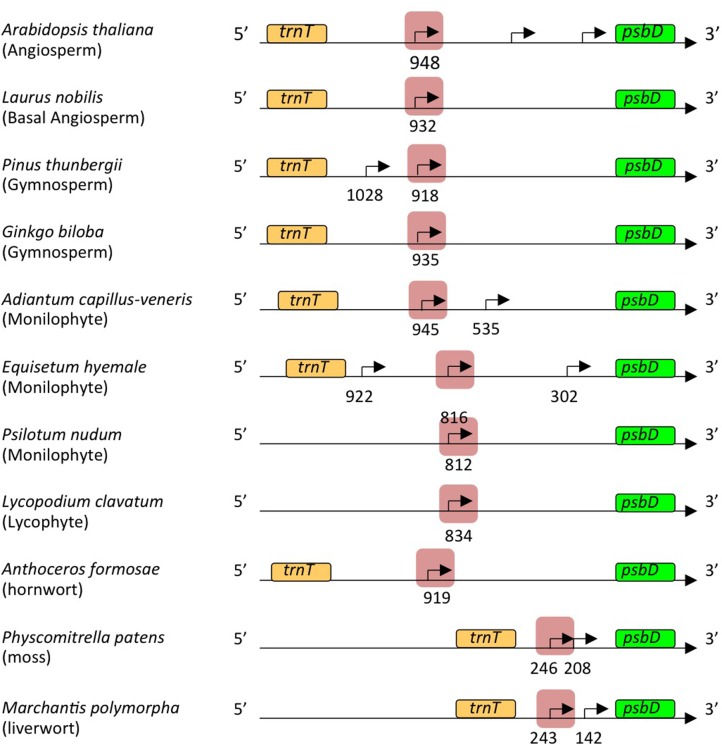
Representative maps of the *psbD* transcripts. The *psbD* transcript 5′-ends identified in **Figure 2** and **Supplementary Figures S1**, **S2** are shown by arrows. The *psbD* LRP-related promoters analyzed in this study are indicated by red shadows.

Transcription initiation sites from the *psbD* LRP have been mapped at 572, 610, 566, 905, and 948 bp upstream of the *psbD* translation start site of barley (Christopher et al., 1992), wheat (Wada et al., 1994), rice (To et al., 1996), tobacco (Yao et al., 1989), and *Arabidopsis* (Hoffer and Christopher, 1997), respectively. Similarly, 5′-ends of the *psbD* primary transcripts of the *psbD-psbC* operon were mapped at 800–900 bp upstream of the *psbD* gene in the most angiosperms including the basal angiosperm *L. nobilis*, gymnosperm *P. thunbergii*, primitive gymnosperm *G. biloba*, monilophyte (Leptosporangiate fern) *A. capillus-veneris*, monilophyte (Eusporangiate fern) *P. nudum*, monilophyte (Eusporangiate fern) *E. hyemale*, and lycophyte *Huperzia lucidula* (**Figure 3**). Furthermore, we found sequences similar to the *psbD* promoter of *H. lucidula* at the far upstream position (-919) of the *psbD* translation start site in *Anthoceros formosae* (hornwort). On the other hand, 5′-ends of the longest *psbD* transcripts of other bryophytes, *P. patens* (moss), and *M. polymorpha* (liverwort) are located at -246 and -243 of the *psbD* gene, respectively. These results indicate that *psbD* mRNAs have unusually long 5′-UTR extensions in common, except for mosses and liverworts. It is of note that intergenic distances between *psbD* and the upstream *trnT* are much shorter in mosses and liverworts compared with those of other plants.

### AAG-Box of the *psbD* LRP Is Highly Conserved among Angiosperms and Gymnosperms, and Partially Conserved in Lycophytes and Mosses

**FIGURE 4:**
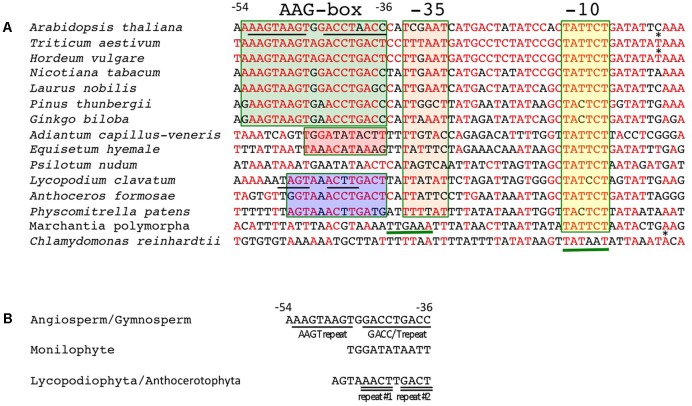
Conserved sequences of the AAG-box containing *psbD* promoters. **(A)** DNA sequences between –54 and +4 of the AAG-box containing *psbD* promoter transcription initiation sites are aligned among the plants analyzed. Transcription initiation sites identified in barley (Kim and Mullet, 1995) and wheat (Nakahira et al., 1998) are indicated by asterisks. Nucleotides that are identical to the wheat sequences are shown in red. The AAG-box in gymnosperms and angiosperms, fern-type upstream sequences, and AAG-box like sequences in basal land plants are indicated by green, red, and blue boxes, respectively. The –35 and –10 elements are indicated by orange and yellow boxes, respectively. The deduced –35 element in *M. polymorpha* sequences and –35 and –10 elements in *C. reinhardtii* are underlined. **(B)** Conserved sequences upstream of the –35 element. Characteristic repeats are underlined.

Next, we compared sequences immediately upstream of the *psbD* transcription initiation sites that were mapped in this and previous studies. The typical -10 elements (TATTCT) are well-conserved in all *psbD* promoters (**Figure 4** and Supplementary Table S2). Conversely, the potential -35 elements are less conserved among terrestrial plants and show weak similarity (less than ∼50%) to the consensus sequences (TTGACA). On the other hand, liverworts possess a typical sigma-70 type promoter with conserved -35 and -10 elements with 18-nt spacing. As expected, the AAG-box is well-conserved among angiosperms and gymnosperms. The consensus sequence of the AAG-box is “RAAGTAAGTRRACCTRACYY,” which contains an AAGT repeat and a GACC/T repeat. The AAG-box sequences are almost 80% identical in most gymnosperms and angiosperms, including the primitive gymnosperm *G. biloba* and basal angiosperm *L. nobilis* (Supplementary Table S2). We found that a 13-bp core sequence of the AAG-box is also highly conserved (∼85%) in lycophytes and hornworts, and partially conserved in mosses (69%), but not in liverworts. The AAG-box of lycophytes and hornworts harbors the GACC/T repeat-like sequences, but lacks the AAGT repeat (**Figure 4B**). On the other hand, neither the AAGT repeat nor GACC/T repeat are conserved in the *psbD* promoter of monilophytes (ferns). Instead, sequences upstream of the -35 element are well-conserved among standard monilophytes and *P. nudum*, but not in the primitive monilophyte *E. hyemale* (**Figure 4B** and **Supplementary Figure S3**). These results suggest that the AAG-box was acquired at a very early stage of embryophyte evolution, and is likely conserved in gymnosperms and angiosperms. On the other hand, monilophytes may have acquired another type of *psbD* promoter with a distinct *cis* element upstream of the -35 element.

### The AAG-Box Containing *psbD* Promoter Is not Associated with Light-Induced *psbD* Expression

**FIGURE 5:**
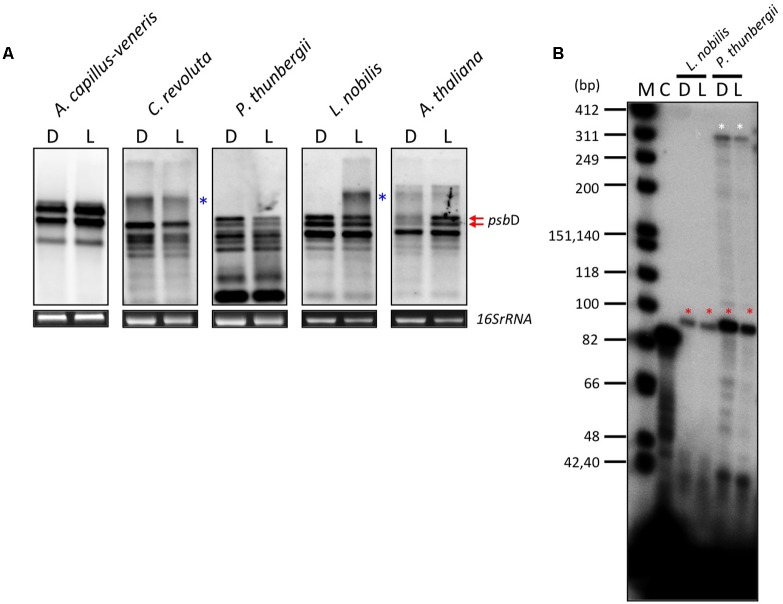
Effects of light on *psbD* transcription. **(A)** DIG-based northern blot analysis of *psbD* transcripts in monilophyte, gymnosperm, and angiosperm. Plants were dark-adapted for 72 h (D) and illuminated for 4 h (180 μmolm^-2^s^-1^; L). Previously characterized transcripts from the AAG-box containing *psbD* promoter are indicated by red arrows in *Arabidopsis*. The smear and extremely large bands (indicated by blue asterisks) represent large read-through transcripts of upstream genes. 16S *rRNA* was used as an RNA-loading control for the total RNA sample. **(B)** The AAG-box containing *psbD* promoter transcripts of *L. nobilis* and *P. thunbergii* were analyzed by primer extension assays. Plants were dark-adapted for 72 h (D) and illuminated for 4 h (180 μmolm^-2^s^-1^; L). Transcripts from the AAG-box containing *psbD* promoter are indicated by red asterisks. The white asterisks show transcripts from the uncharacterized promoter upstream of the AAG-box containing *psbD* promoter in *P. thunbergii*. Lane C shows an experiment with the control RNA and primer provided by the manufacturer that produces an 87-base primer extension product.

In order to address whether the *psbD* LRP is responsible for light-induced transcription, we examined the light-induced expression of *psbD* transcripts in some embryophytes, including monilophytes, gymnosperms, and angiosperms. As shown in **Figure 5A**, *psbD* expression is clearly induced by light in *A. thaliana* (angiosperm). However, unexpectedly, no light-induced psbD expression was detected in *L. nobilis* (basal angiosperm), *P. thunbergii* (gymnosperm), or *Cycas revoluta* (primitive gymnosperm), although they all have a well-conserved AAG-box containing *psbD* promoter. Similarly, *psbD* expression is also not regulated by light in *A. capillus-veneris* (monilophyte), which does not have the conserved AAG-box in the *psbD* promoter. We further examined the light-mediated regulation of the AAG-box containing *psbD* promoter transcripts by primer extension analysis. As shown in **Figure 5B**, the abundance of the transcripts from the AAG-box containing *psbD* promoter was not regulated by light in *L. nobilis* or *P. thunbergii* (**Figure 5B**). These results suggest that the AAG-box containing *psbD* promoter is not directly associated with light-induced *psbD* expression.

Moreover, we examined light- and osmotic stress-induced *psbD* expression in a wide range of plants. In angiosperms, light-induced *psbD* expression was detected in a number of eudicots (*C. sativus*, *A. thaliana*, and *L. sativa*) and monocots (wheat and maize), whereas *psbD* expression is not regulated by light in basal angiosperms except for *C. glaber* (**Supplementary Figures S4**–**S6**). It is to be noted that *psbD* expression is activated by long-term illumination (12 h) in *C. glaber* (**Supplementary Figure S6**). In contrast, the osmotic stress-induced expression of *psbD* was detected only in eudicots. Expression of *psbD* was not activated by osmotic stress in monocots and basal angiosperms except for *C. glaber* (**Supplementary Figure S5**, **S6**). Furthermore, neither light nor osmotic stress-induced *psbD* expression was detected in gymnosperms, *Gingko* and *Cycas* (**Supplementary Figure S6**). Although psbD expression was not activated by moderate light (180 μmolm^-2^s^-1^) in *Pinus* (**Figure 5**), high light exposure (245 μmolm^-2^s^-1^) induced transient expression of *psbD* (**Supplementary Figure S6**). It is of note that gymnosperms and basal angiosperms have a well-conserved AAG-box containing *psbD* promoter. On the other hand, *psbD* expression is induced by light and/or osmotic stress in some monilophytes that lack the typical AAG-box containing *psbD* promoter. In addition, light and salt stress barely affect *psbA* expression in any of the plants examined (**Supplementary Figures S5**–**S8**). These results indicate that light and salt stress-induced transcription has evolved independently and multiple times during land plant evolution. Furthermore, the AAG-box in the *psbD* promoter is unlikely to be directly associated with the occurrence of light and salt stress-induced *psbD* expression.

### Gnetaceae Plants in Gymnosperms Have Lost the AAG-Box Containing *psbD* Promoter

**FIGURE 6:**
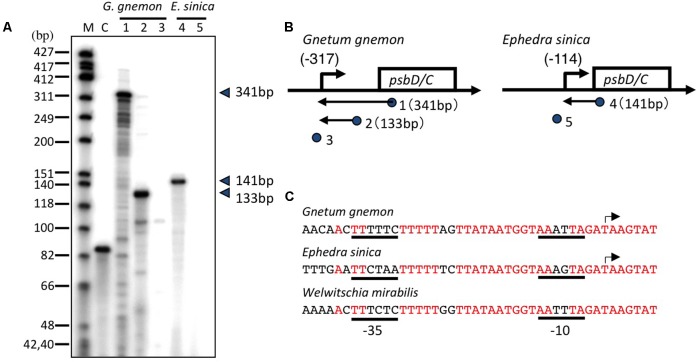
Mapping of the 5′ ends of the *psbD* transcripts in Gnetaceae plants. **(A)** The *psbD LRP* transcripts of the Gnetaceae plants *G. gnemon* (lanes 1–3) and *E. sinica* (lanes 4 and 5) were analyzed by primer extension assays. Primers used are indicated by numbers on the top of each lane. The size of the extension product is shown on the right. Lane C shows an experiment with the control RNA and primer provided by the manufacturer that produces an 87-base primer extension product. **(B)** Representative maps of the *psbD* transcripts. The *psbD* transcript 5′-ends identified by the primer extension assay are shown by arrows. The position of primers and the size of the extension products are shown on the gene map. The deduced sites of the 5′-end of each transcript are shown as numbers in parentheses. No transcript was detected with #3 and #5 primers. **(C)** DNA sequences between –37 and +7 of the AAG-box containing *psbD* promoter transcription initiation sites of *G. gnemon* and *E. sinica* are shown. The corresponding sequences of the *psbD* upstream region of *Welwitschia mirabilis* are also shown. Transcription initiation sites are indicated by arrows. Potential –35 and –10 elements are underlined.

Gnetaceae plants are a unique group of gymnosperms that have evolved a morphological system related to that of the angiosperms. The most upstream transcription initiation sites have been mapped at 317 and 114 bp upstream of the *psbD* translation start site of *Gnetum gnemon* and *Ephedra sinica*, respectively (**Figures 6A,B**). The *psbD* transcripts of gnetaceae have shorter 5′-UTR compared with the standard *psbD* transcripts in other plants. The upstream sequences of the *psbD* transcripts are well-conserved among gnetaceae. No AAG-box-like sequences are found upstream of the *psbD* transcripts in gnetaceae, whereas the gnetaceae *psbD* promoters possess -35- and -10-like sequences (**Figure 6C**). The similar sequences are also found upstream of the psbD-C operon of *Welwitschia mirabilis*. Moreover, AAG-box-like sequences have not been identified in the *trnT-psbD* intergenic regions of gnetaceae. We suggest that the AAG-box containing *psbD* promoter was lost in gnetaceae during their evolution. It would be very interesting to determine whether *psbD* expression is dependent on light in gnetaceaes.

## Discussion

The *psbD* and *psbC* genes are organized in a *psbD-psbC* operon, which is well-conserved among plants and cyanobacteria. Transcription from the *psbD-psbC* operon is mediated solely by PEP in green tissues. Multiple transcriptional start sites (TSS) generating mRNAs with heterogeneous 5′ transcript leaders have been mapped on the *psbD-psbC* operon in several angiosperm plants, including tobacco (Yao et al., 1989), barley (Christopher et al., 1992), wheat (Wada et al., 1994), rice (To et al., 1996), and *Arabidopsis* (Hoffer and Christopher, 1997). Light activates the expression of some *psbD-psbC* mRNAs, whereas the accumulation of other mRNAs is not regulated by light. Analysis of the promoter sequences immediately upstream of the light-induced TSS identified an unusual PEP promoter consisting of a core promoter with a weakly conserved -35 element along with an upstream *cis* element termed the AAG-box (**Figure 1A**). This promoter is specifically activated by high-irradiance blue light and UV-A (Christopher and Mullet, 1994), and is designated as the *psbD* light-responsive promoter (*psbD* LRP) or *psbD* blue light-responsive promoter (*psbD* BLRP). As shown in **Figure 1B**, the *psbD* LRP is also activated by various environmental stresses (Nagashima et al., 2004). On the other hand, other light-insensitive promoters mapped on the *psbD-psbC* operon are standard PEP promoters composed of well-conserved -10 and -35 elements.

In order to investigate *psbD LRP* evolution, we mapped promoters responsible for the expression of the *psbD-psbC* operon by primer extension analysis in 11 embryophytes at different evolutionary stages from liverworts to angiosperms. We considered the *psbD* transcripts with the most upstream terminus as the primary transcripts, except for *P. thunbergii* and *E. hyemale*, which have another promoter upstream of the potential AAG-box containing *psbD* promoter. All *psbD* promoters identified at the most upstream mRNA terminus have the well-conserved -10 element (TATTCT) that is similar to the standard -10 element (TATAAT). On the other hand, the potential -35 elements of the *psbD* promoters are less conserved, suggesting the limited role of the -35 element in *psbD* promoter activity. *In vitro* transcription experiments have demonstrated that the -10 element is important for transcription from the AAG-box containing *psbD* promoter, but the -35 element is not essential for transcription in barley, rice, or wheat (To et al., 1996; Nakahira et al., 1998; Kim et al., 1999). It is considered that the -10 element is important for *psbD* promoter activity, but not the poorly conserved -35 element.

We found that the 19 bp AAG-box sequences of the *psbD* promoters are highly conserved among gymnosperms and angiosperms, including the basal angiosperm *L. nobilis* and primitive gymnosperm *G. biloba*. The consensus AAG-box sequence is “RAAGTAAGTRRACCTRACY,” which is at least 80% identical in gymnosperms and angiosperms. The AAG-box is composed of two repeat sequences: AAGT and GACC/T repeats. Extensive analyses of the AAG-box containing *psbD* promoter structure using *in vitro* transcription systems have revealed that both the AAGT and GACC/T repeats are important for AAG-box containing *psbD* promoter activity (Kim and Mullet, 1995; To et al., 1996; Nakahira et al., 1998). Moreover, the AAG-box is also partially conserved in basal land plants such as lycophytes, hornworts, and mosses. We identified the shorter conserved AAG-box-like sequences in *H. lucidula* (lycophyte) and *A. formosae* (hornwort). The AAG-box-like sequences in lycophyta and hornworts retain a partially conserved GACC/T repeat, but lack AAGT repeats. Deletion of the AAGT repeat resulted in only a partial reduction of *in vitro* transcription activity of the AAG-box containing *psbD* promoter in wheat, suggesting that the GACC/T repeat is sufficient to mediate AAG-box containing *psbD* promoter activity (Nakahira et al., 1998). Thus, the AAG-box-like sequences may act as a transcription activation element in the *psbD* promoters of basal land plants. Considering the highly conserved -10 element among bryophytes and angiosperms, it is likely that the last common ancestor of bryophytes and spermatophytes likely already possessed an AAG box-containing *psbD* LRP (**Supplementary Figure S9**). The AAG box may have developed to take over the function of the -35 element and support the high-level transcription activity of the AAG-box containing *psbD* promoter.

On the other hand, the AAG-box like sequences have not been identified in the *psbD* promoter of *M. polymorpha* (liverwort). The liverwort *psbD* promoter is composed of the typical -35 element (TTGAAA) and the -10 element (TATTCT) with standard spacing, suggesting that *psbD* is transcribed from a standard PEP promoter in liverworts. It is to be noted that the *psbD* promoter has a well-conserved -10 element, but lacks the AAG-box and standard -35 element in *Chlamydomonas* (Klein et al., 1992; Klinkert et al., 2005).

Monilophytes (ferns) have another type of *psbD* promoter that lacks the conserved AAG-box. Instead, 11-bp sequences upstream of the potential -35 element are well-conserved among standard (*A. capillus-veneris*) and primitive (*P. nudum*) monilophytes. However, it remains elusive whether the conserved sequences upstream of the psbD transcription stat site in monilophytes is required for *psbD* transcription. Interestingly, gnetaceae plants in gymnosperms have lost the AAG-box-containing *psbD* LRP, suggesting that the AAG-box containing *psbD* promoter is not essential for plant development.

It has also been shown that an AAG-box-binding factor (AGF) specifically binds to the AAGT repeat, that it is partially associated with the GACC/T repeats (Kim and Mullet, 1995; Kim et al., 1999), and that it activates transcription from the AAG-box containing *psbD* promoter (Wada et al., 1994; Kim and Mullet, 1995; Allison and Maliga, 1995; To et al., 1996; Nakahira et al., 1998; Kim et al., 1999). PTF1 (plastid transcription factor 1) is a basic helix-loop-helix DNA-binding protein, which binds to the AAG box and is involved in transcription from the *psbD* LRP in *Arabidopsis* (Baba et al., 2001). However, close orthologs of the *Arabidopsis* PTF1 have only been found in angiosperms, suggesting that PTF1 is responsible for AAG-box-dependent transcription at the AAG-box containing *psbD* promoter in angiosperms. However, the role of AGF in the light-dependent transcription remains to be elucidated.

In addition, reverse genetic analysis of sigma factors revealed that the AAG-box containing *psbD* promoter is specifically recognized by SIG5 in *Arabidopsis* (Nagashima et al., 2004; Tsunoyama et al., 2004). Transcription from the AAG-box containing *psbD* promoter is likely to be mediated by SIG5 containing PEP and activated by AGF that binds to the AAG-box. However, overexpression of SIG5 cannot activate transcription from the AAG-box containing *psbD* promoter in the dark (**Figure 1C**). Furthermore, photoreceptors including CRY1, CRY2, and PhyA are involved in the light-induced expression of the *psbD-psbC* operon in *Arabidopsis* (Thum et al., 2001a). Taken together, photoreceptor-mediated signaling may modify SIG5 or SIG5 import into the chloroplasts in a light-dependent manner and activate transcription at the AAG-box containing *psbD* promoter.

*SIG5* orthologs have been identified in a number of angiosperms. Moreover, SIG5 has been identified as a gene that was abundant when water availability was low in gymnosperm *Pseudotsuga menziesii* (Hess et al., 2016). Furthermore, SIG5 orthologs have been identified in *P. patens* (Ichikawa et al., 2008) and *Selaginella moellendorffii* (XP_002970534). It has been shown that PpSIG5 is involved in high-intensity light and circadian control of *psbD* expression in the moss *P. patens* (Ichikawa et al., 2004, 2008). Taken together, these results suggest that SIG5 plays a role in transcription from the *psbD* promoter consisting of the AAG-box or AAG-like element in embryophytes including mosses. On the other hand, an *SIG5* ortholog was also identified in the liverwort *M. polymorpha* (Kanazawa et al., 2013). MpSIG5 is not necessary for light-dependent *psbD* expression in *M. polymorpha* (Kanazawa et al., 2013). MpSIG5 may have another role in chloroplast transcription in *M. polymorpha* that lacks the AAG-box containing *psbD* promoter. Furthermore, *SIG5* orthologs have been identified in some charophytes such as *K. flaccidum*, but not in the green alga *C. reinhardtii* or in the primitive red alga *C. merolae*. It is likely that SIG5 was acquired in charophytes before the occurrence of AAG-box containing *psbD* promoter in basal embryophytes.

Transcripts from the AAG-box containing *psbD* promoter are characterized by a markedly long 5′-UTR. All AAG-box containing *psbD* promoter transcripts except for *P. patens* and *M. polymorpha* have a 5′-UTR longer than 500 nucleotides from the translation start site. Detailed mapping of the *psbD* transcripts in angiosperm plants revealed that no intron is present in the 5′-UTR and the AAG-box containing *psbD* promoter transcripts actually have a long 5′-UTR (Wada et al., 1994; Hoffer and Christopher, 1997; Kim et al., 1999). The 5′-UTR of chloroplast transcripts may be involved in the stability of the transcripts and/or efficiency of translation (Shiina et al., 1998). However, no conserved sequences have been identified in the 5′-UTR of embryophytes. Further characterization of the 5′-UTR would shed light on the role of the unusually long 5′-UTR of the AAG-box containing *psbD* promoter in most embryophytes,

This study suggest that the AAG-box containing *psbD* promoter appeared in basal embryophytes more than 450 million years ago, and the common ancestor of bryophytes and spermatophytes likely possessed an AAG box-containing *psbD* promoter. On the other hand, the -35 and -10 elements of the *psbA* and *rbcL* promoters are almost identical among liverworts and angiosperms (**Supplementary Figure S10**). It is suggested that ecological and/or physiological demands may have accelerated the evolution of the AAG-box containing *psbD* promoter in embryophytes. One of the unique characteristics of the AAG-box containing *psbD* promoter is light- and stress-induced transcription. However, extensive expression analysis of *psbD* in a variety of plants revealed that the light and/or stress-induced expression of the *psbD* gene developed independently in several plants. Thus, it is unlikely that that light and/or stress responses of the AAG-box containing *psbD* promoter are directly associated with AAG-box containing *psbD* promoter evolution. Recent studies demonstrated that ABA and the circadian rhythm regulate chloroplast AAG-box containing *psbD* promoter activity via the activation of SIG5 (Noordally et al., 2013; Yamburenko et al., 2015; Belbin et al., 2017) Further analysis of the role of the AAG-box containing *psbD* promoter in chloroplasts may shed light on AAG-box containing *psbD* promoter evolution.

## Author Contributions

TS, SS, SM, and YN designed research. SS, MN, ShK, SaK, and YI performed research. TS, SS, and MN analyzed data. TS, SS, and MN wrote the paper. SS and MN have contributed equally to this work.

## Conflict of Interest Statement

The authors declare that the research was conducted in the absence of any commercial or financial relationships that could be construed as a potential conflict of interest.

## Supplementary Material

**FIGURE S1 |** Mapping of the 5′ ends of the *psbD* transcripts *psbD* transcripts were isolated from liverwort *M. polymorpha* **(A)**, lycophyte *H. lucidula* **(B)**, and monilophyte *P. nudum* **(C)**. Primers used are indicated by numbers on the top of each lane. The size of the extension product is shown on the right. The position of primers and the size of the extension products are shown on the gene map. The deduced sites of the 5′-end of each transcript are shown as numbers in parentheses. Lane Xs show the results of unrelated samples. Lane C shows an experiment with the control RNA and primer provided by the manufacturer that produces an 87-base primer extension product.

**FIGURE S2 |** Mapping of the 5’ ends of the *psbD* transcripts *psbD* transcripts were isolated from monilophyte *E. hyemale* **(A)**, gymnosperm *P. thunbergii* **(B)**, and angiosperm *L. nobilis* **(C)**. Primers used are indicated by numbers on the top of each lane. The size of the extension product is shown on the left in **(A)** and on the right in **(B,C)**. The position of primers and the size of the extension products are shown on the gene map. The deduced sites of the 5′-end of each transcript are shown as numbers in parentheses. Lane C shows an experiment with the control RNA and primer provided by the manufacturer that produces an 87-base primer extension product.

**FIGURE S3 |** A comparison of the potential *psbD* promoter sequences in monilophytes DNA sequences upstream of the *psbD* transcription initiation site of *A. capillus-veneris* and corresponding sequences of three other leptosporangiate ferns are compared with the *psbD* promoter sequences of two primitive ferns (*P. nudum* and *E. hyemale*). Transcription initiation sites identified in this study are indicated by asterisks. Nucleotides that are identical to the *Adiantum* sequences are shown in red. The monilophytes-type upstream conserved sequences are indicated by a green box. The -35 and -10 elements are indicated by orange and yellow boxes, respectively.

**FIGURE S4 |** Northern blot analysis of *psbD* and *psbA* transcripts in eudicots. Plants were dark-adapted for 72 h (D) and illuminated for up to12 h (275 μmolm^-2^s^-1^; L). Osmotic stress was applied by 250 mM mannitol for 6 h. Light- or salt stress-induced *psbD* transcripts are indicated by red arrow heads. rRNA stained with EtBr was used as an RNA-loading control for the total RNA sample.

**FIGURE S5 |** Northern blot analysis of *psbD* and *psbA* transcripts in monocots. Plants were dark-adapted for 72 h (D) and illuminated for up to12 h (275 μmolm^-2^s^-1^; L). Osmotic stress was applied by 250 mM mannitol for 6 h. Light- or salt stress-induced *psbD* transcripts are indicated by red arrow heads. rRNA stained with EtBr was used as an RNA-loading control for the total RNA sample.

**FIGURE S6 |** Northern blot analysis of *psbD* and *psbA* transcripts in basal angiosperms. Plants were dark-adapted for 72 h (D) and illuminated for 12 h (275 μmolm^-2^s^-1^; L). Osmotic stress was applied by 250 mM mannitol for 6 h. Light- or salt stress-induced *psbD* transcripts are indicated by red arrow heads. rRNA stained with EtBr was used as an RNA-loading control for the total RNA sample.

**FIGURE S7 |** Northern blot analysis of *psbD* and *psbA* transcripts in gymnosperms. Plants were dark-adapted for 72 h (D) and illuminated for up to 12 h (275 μmolm^-2^s^-1^; L). Osmotic stress was applied by 250 mM mannitol for 6 h. Light- or salt stress-induced *psbD* transcripts are indicated by red arrow heads. rRNA stained with EtBr was used as an RNA-loading control for the total RNA sample.

**FIGURE S8 |** Northern blot analysis of *psbD* and *psbA* transcripts in monilophytes. Plants were dark-adapted for 72 h (D) and illuminated for up to 12 h (275 μmolm^-2^s^-1^; L). Osmotic stress was applied by 250 mM mannitol for 6 h. Light- or salt stress-induced *psbD* transcripts are indicated by red arrow heads. rRNA stained with EtBr was used as an RNA-loading control for the total RNA sample.

**FIGURE S9 |** Phylogenetic tree of AAG box. Alignments were undertaken with 19 bps sequences (from -54 to -36 of the transcription initiation site) of 18 plants from *C. reinhardtii* to *A. thaliana*.

**FIGURE S10 |** Comparion of *psbA* and *rbcL* promoters of terrestrial plants.
