# Supplementary figures and images for: Comparative Analysis of Chloroplast psbD Promoters in Terrestrial Plants
Source: Front Plant Sci. 2017 Jul 13;8:1186. doi: 10.3389/fpls.2017.01186 (PMC5508017; doi:10.3389/fpls.2017.01186)

sFig. 1

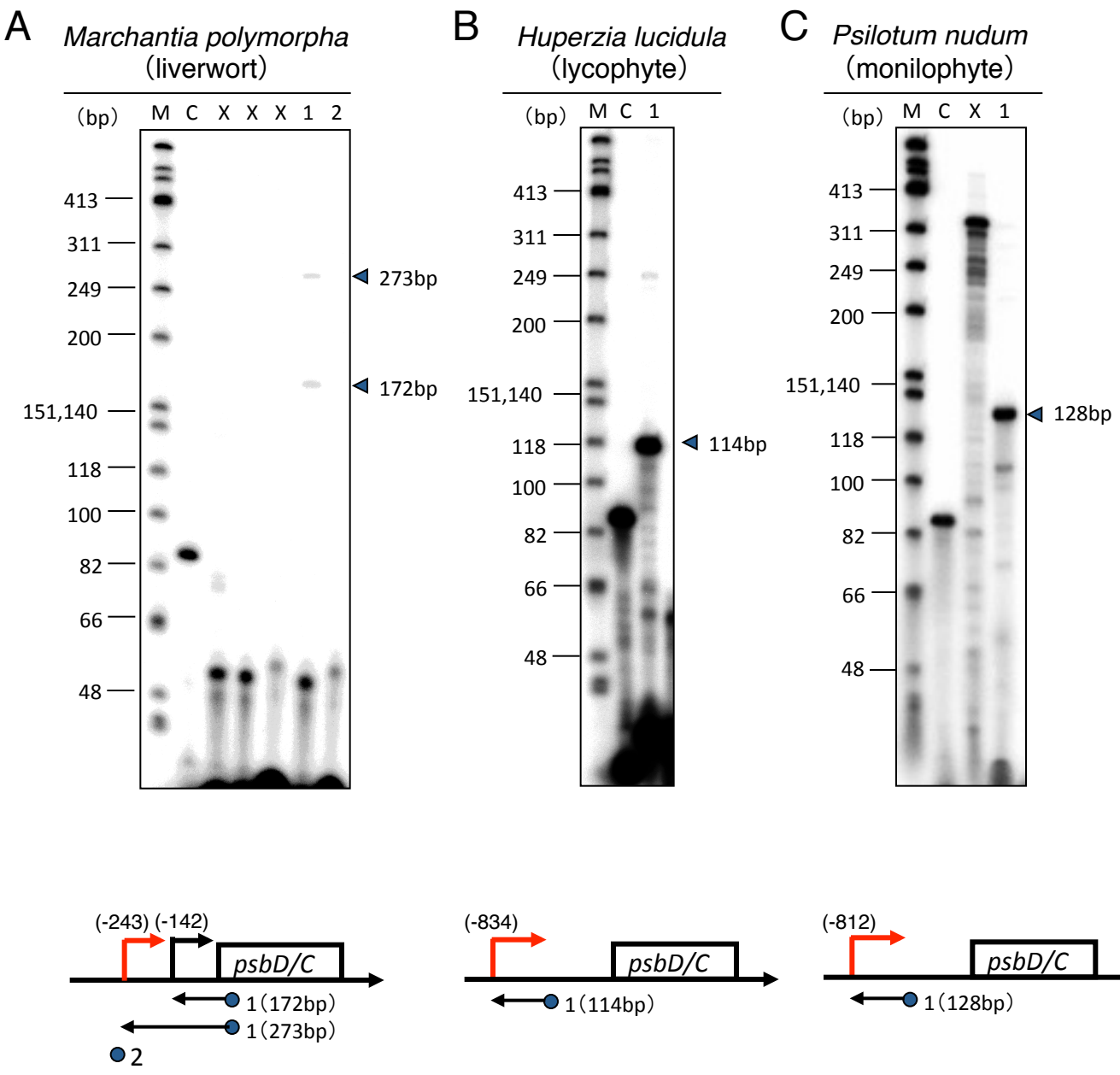

Supplement: Supplementary file 1 [file Presentation_1.pdf]

sFig. 3

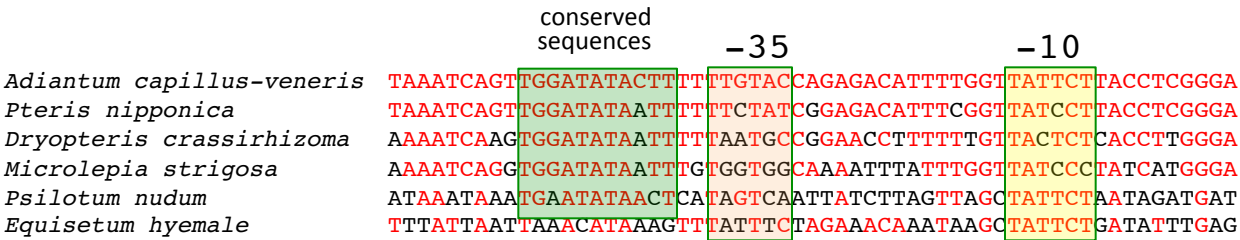

Supplement: Supplementary file 3 [file Presentation_3.pdf]

sFig. 4

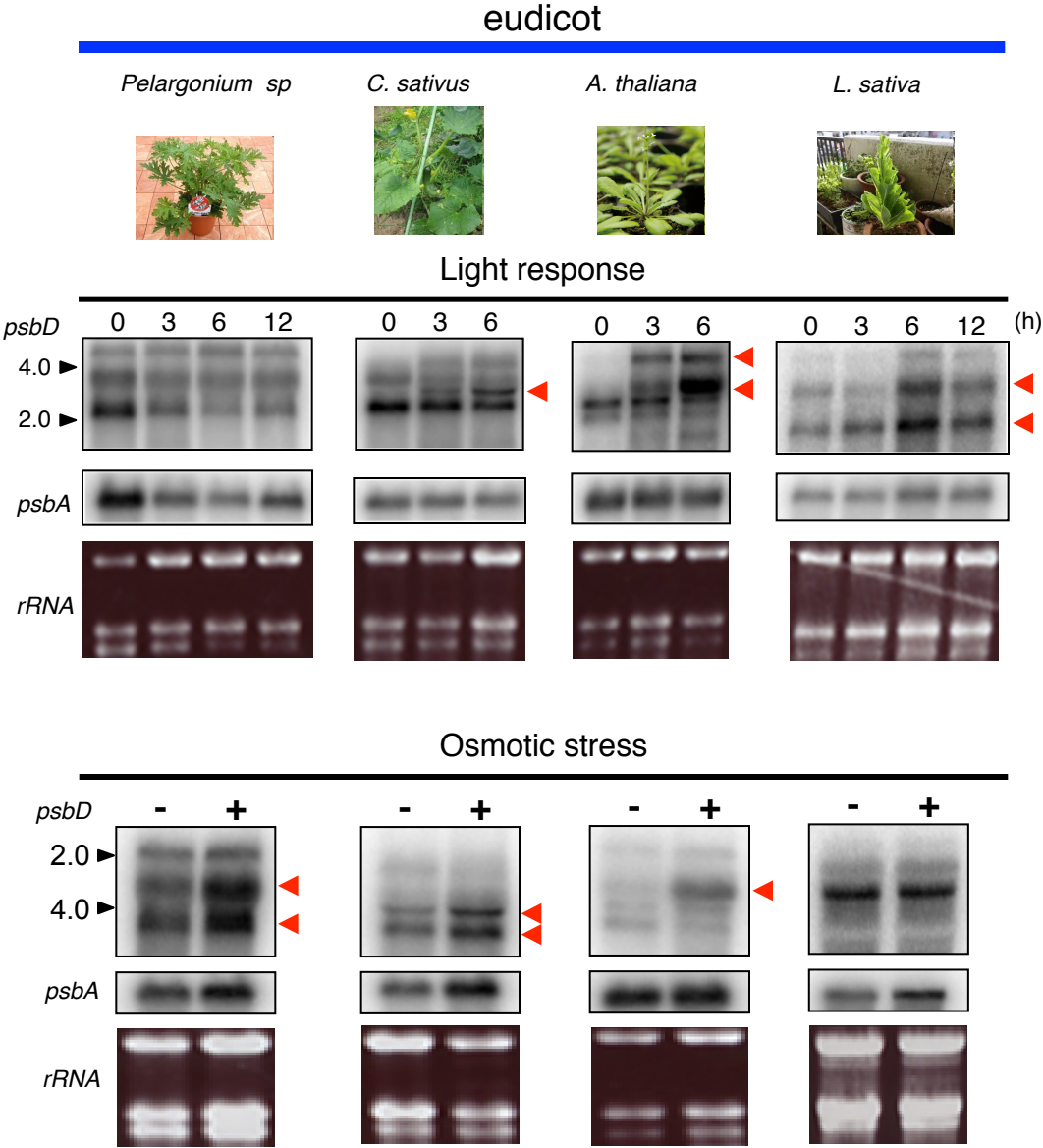

Supplement: Supplementary file 4 [file Image_4.pdf]

sFig. 5

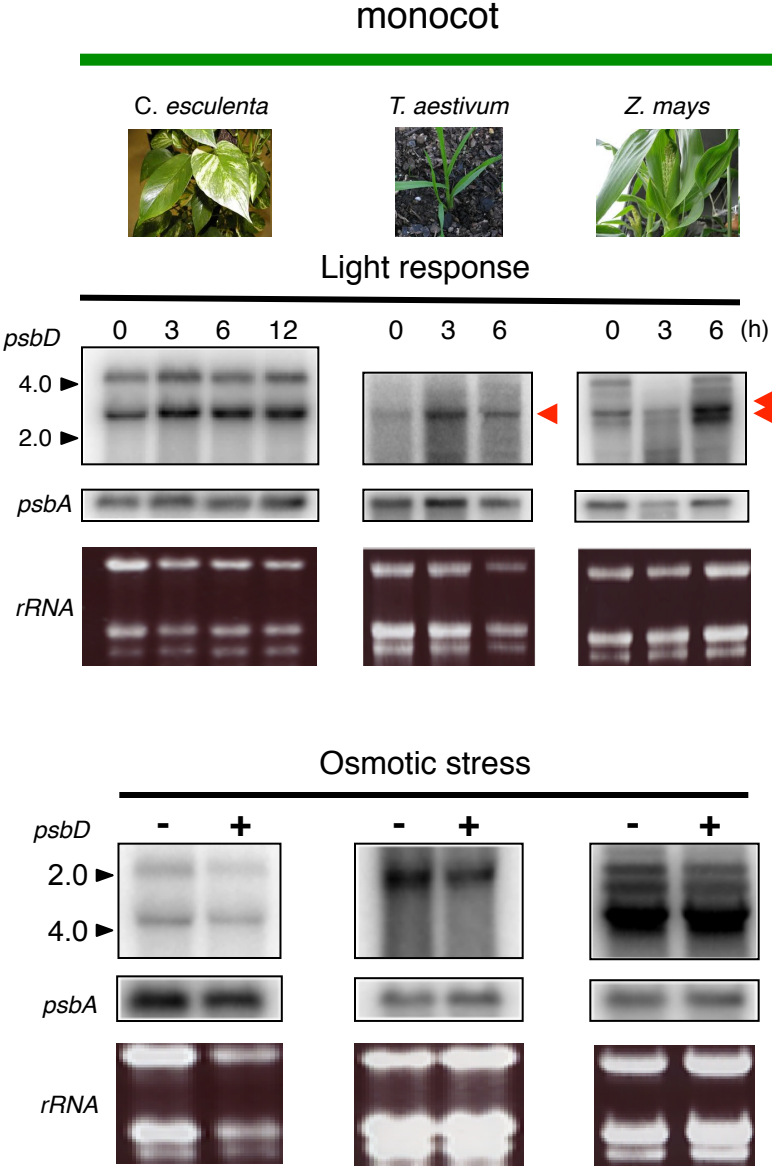

Supplement: Supplementary file 5 [file Image_5.pdf]

sFig. 6

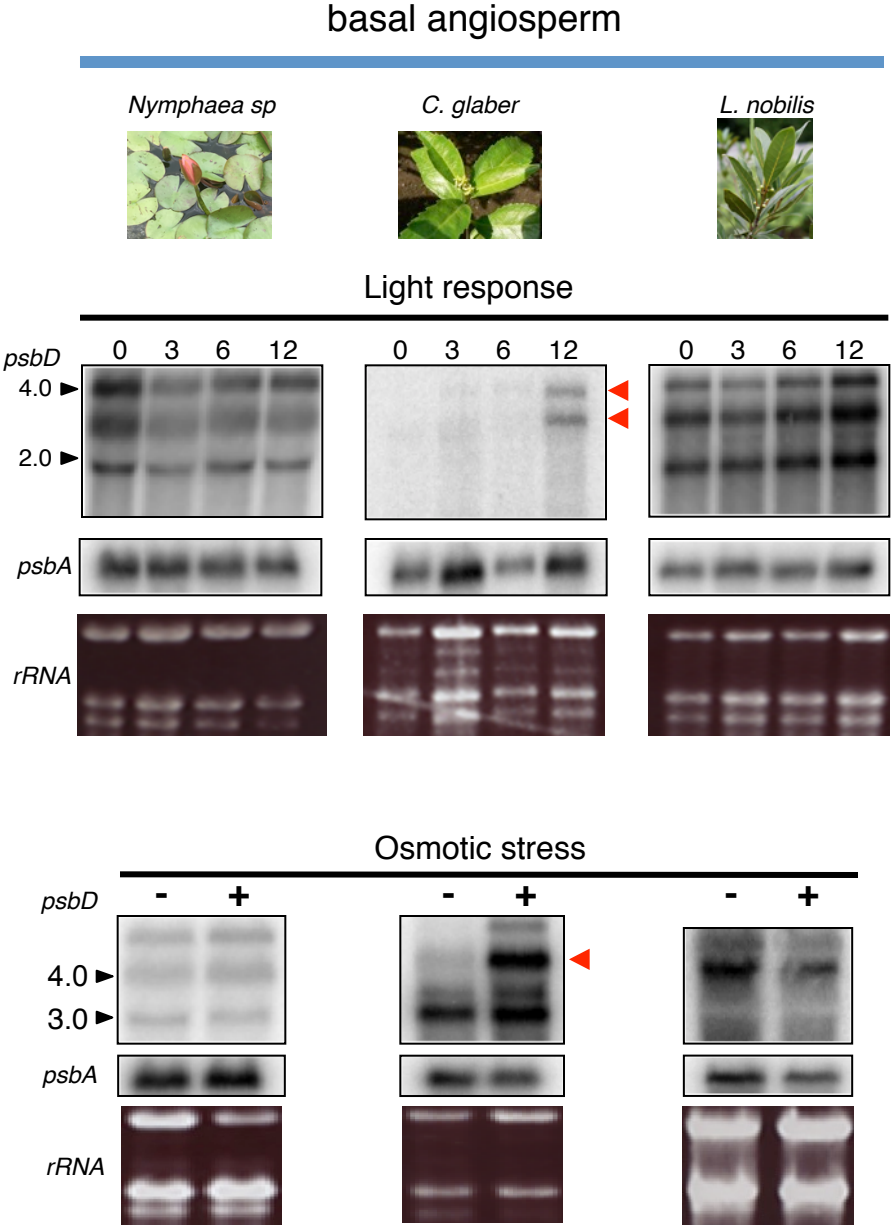

Supplement: Supplementary file 6 [file Presentation_6.pdf]

sFig. 7

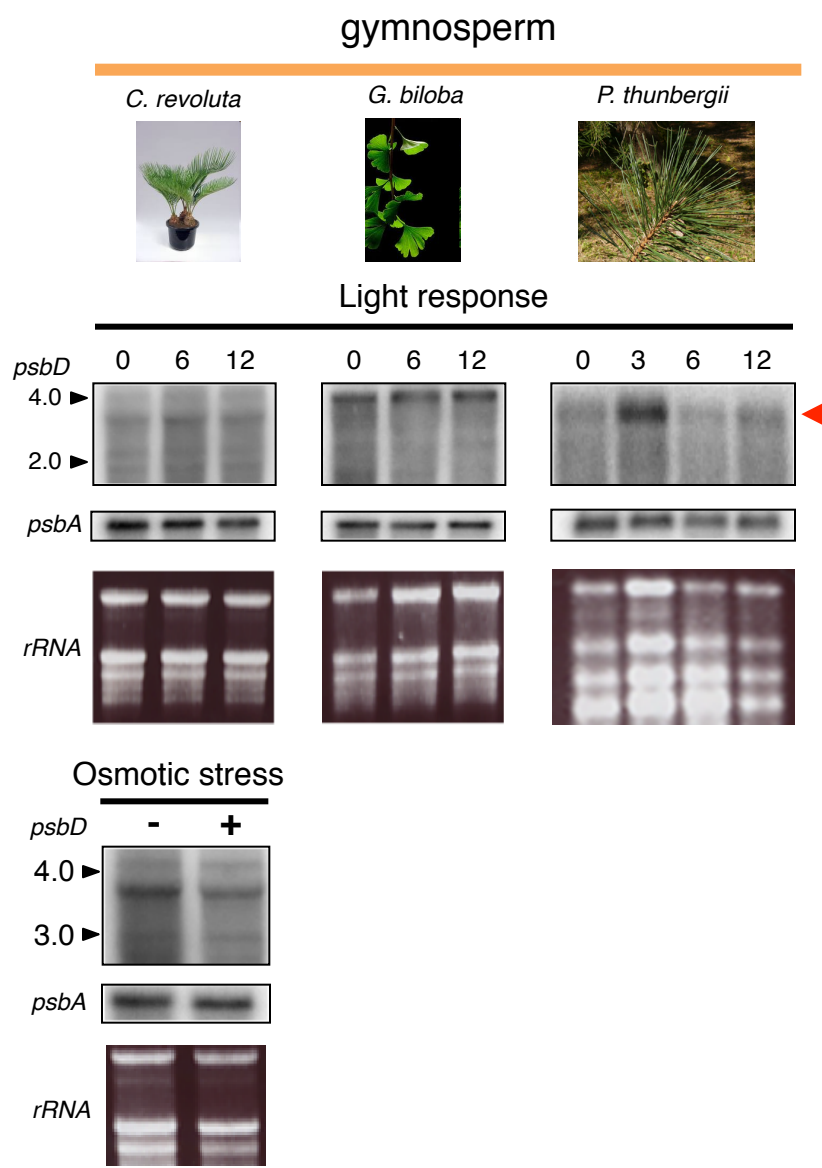

Supplement: Supplementary file 7 [file Presentation_7.pdf]

sFig. 8

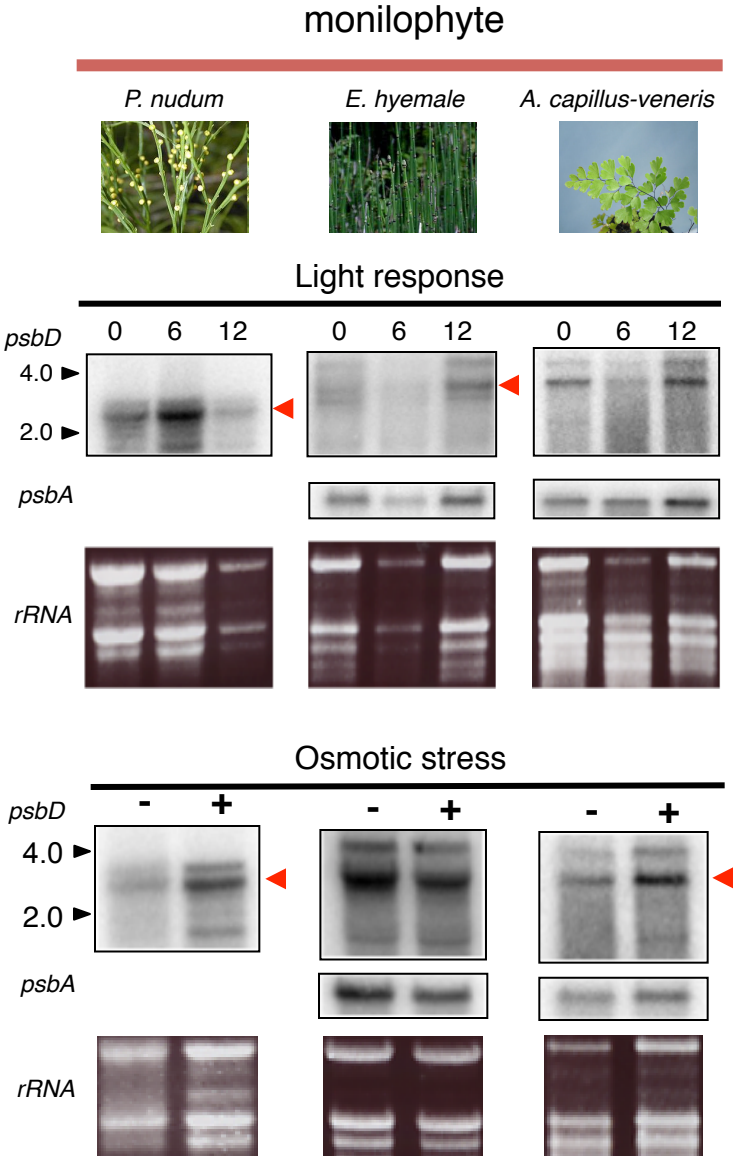

Supplement: Supplementary file 8 [file Presentation_8.pdf]

sFig.9

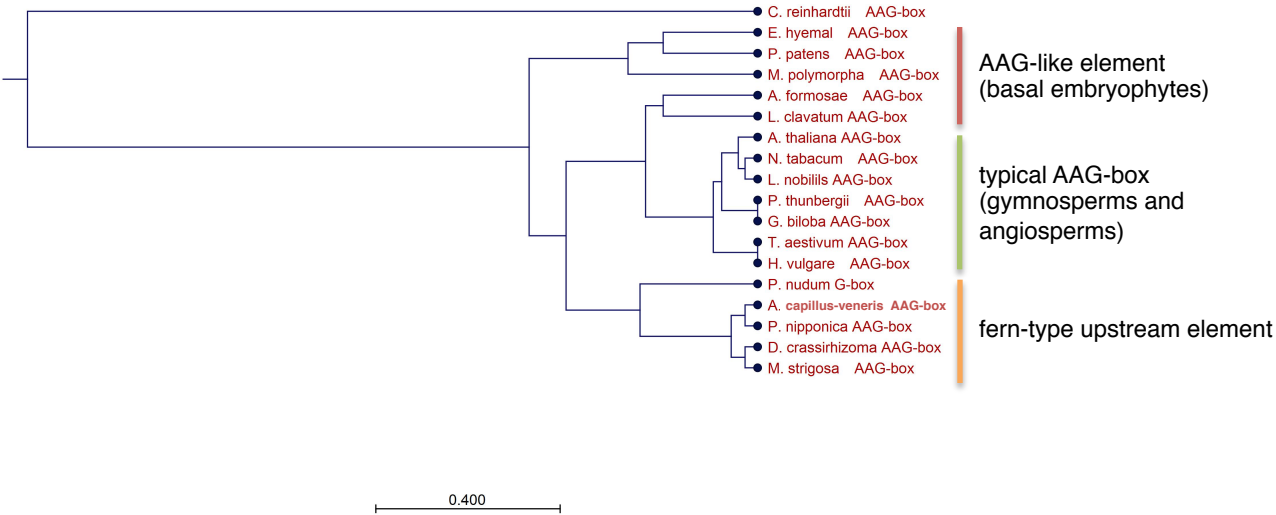

Supplement: Supplementary file 9 [file Presentation_9.pdf]
