# Supplementary material for: Comparative Analysis of Chloroplast psbD Promoters in Terrestrial Plants
Source: Front Plant Sci. 2017 Jul 13;8:1186. doi: 10.3389/fpls.2017.01186 (PMC5508017; doi:10.3389/fpls.2017.01186)
Supplement: Supplementary file 2 [file Image_2.pdf]

sFig. 2

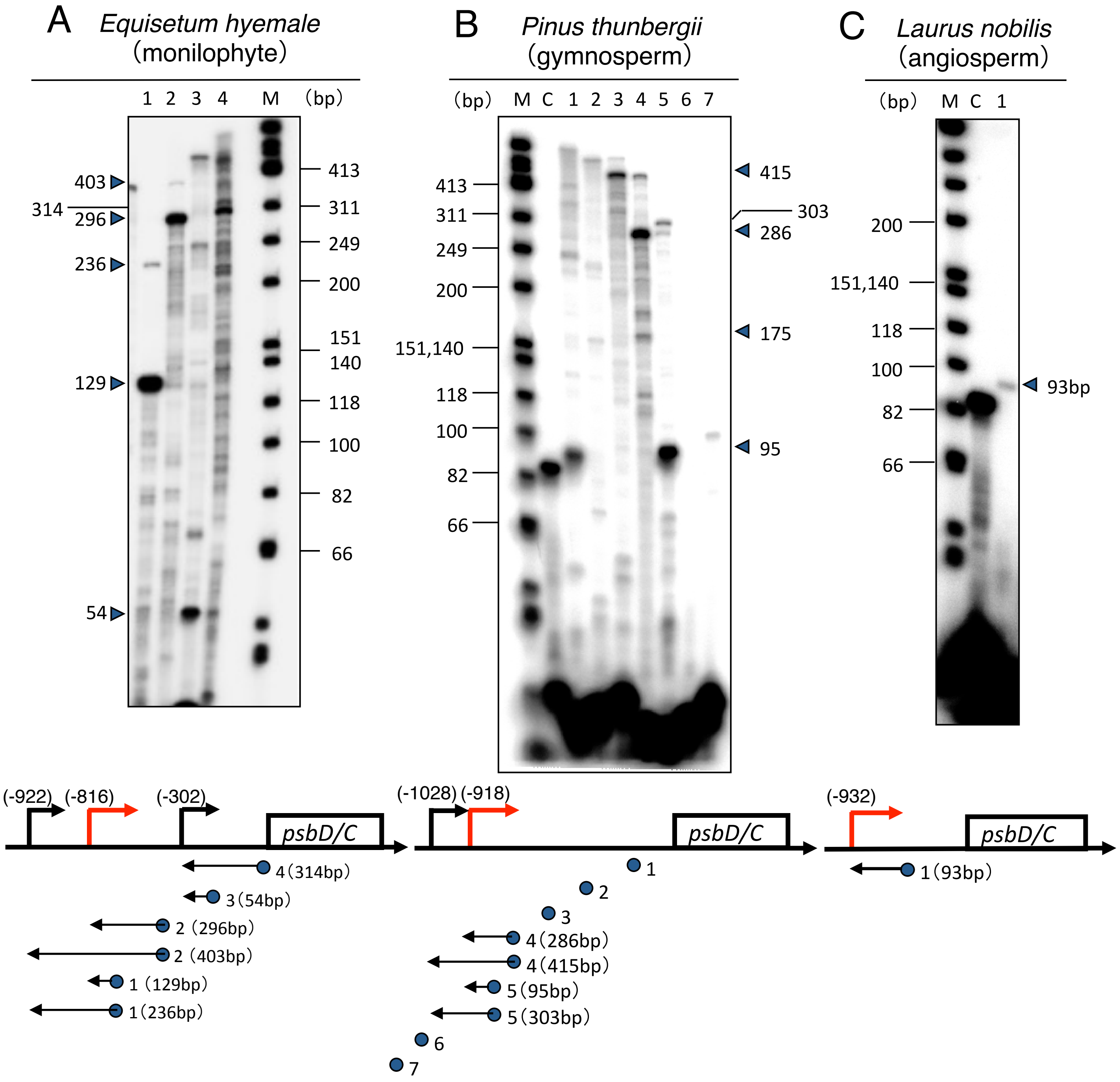
A *Equisetum hyemale* (monilophyte)
1 2 3 4 M (bp)
403
314
296
236
129
54
413
311
249
200
151
140
118
100
82
66
(-922) (-816) (-302)
psbD/C
4 (314bp)
3 (54bp)
2 (296bp)
2 (403bp)
1 (129bp)
1 (236bp)
B *Pinus thunbergii* (gymnosperm)
(bp) M C 1 2 3 4 5 6 7
413
311
249
200
151,140
118
100
82
66
415
303
286
175
95
(-1028) (-918)
psbD/C
1
2
3
4 (286bp)
4 (415bp)
5 (95bp)
5 (303bp)
6
7
C *Laurus nobilis* (angiosperm)
(bp) M C 1
200
151,140
118
100
82
66
93bp
(-932)
psbD/C
1 (93bp)
