# Supplementary material for: Comparative Analysis of Chloroplast psbD Promoters in Terrestrial Plants
Source: Front Plant Sci. 2017 Jul 13;8:1186. doi: 10.3389/fpls.2017.01186 (PMC5508017; doi:10.3389/fpls.2017.01186)
Supplement: Supplementary file 10 [file Image_10.pdf]

sFig.10

psbA promoter

```
T. aestivum           TTGGTTGACATTGGTATATAGTCTATGTTATACTGTTAA
O. sativa             TTGGTTGACATTGGTATATAGTCTATATTATACTGTTAA
A. thaliana           TTGGTTGACATGGCTATATAAGTCATGTTATACTGTTTC
N. tabacum            TTGGTTGACACGAGTATATAAGTCATGTTATACTGTTGA
N. alba               TTAGTTGACACGGGCATATAAGGCATGTTATACTGTTGA
A. trichopoda         TTGGTTGACACAGGCATATAGGTCATGTTATACTGTTGA
P. thunbergii         TTGGTTGACATTGATACATGGATCATATTATACTGTAAA
C. taitungensis       TCGATTCACGATA-TATATAAGTCATACTATACTGTTAA
W. mirabilis          ATAGTTGACTTTAATAAACCATTTCTGTTATACTGTTAA
A. evecta             TAAGTTGACATCAATAGATAAGTTGTGTTATACTATGAA
A. capillus-veneris   TTGGTTGACACGGATAGGTTTT-TGTGATATGCTACATA
P. nudum              TAAGTTGACATATATGGAAAGATCATGTTATACTTCAAA
H. lucidula           TGGGTTGACACAAAAAGAAAGATTGTGTAATATTATGGA
P. patens             TCAGTTGACATAATAATACATTTTGTGTAATACTATAAA
M. polymorpha         TTAGTTGACATAATCATATGTTATGTGTAATACTATAAG
C. vulgaris           CTAGTTGACATTTTTATACTTTACATACTATAATATCTA
                             -35        TATA          -10
                           TTGACA       TATA        TATACT
```

rbcL promoter

```
T. aestivum           TGGGTTGCGCTATATCTATCAAAGAGTATACAATAATTA
O. sativa             GGGGTTGCGCTATATCTATTAAAGAGTATACAATAAAGA
A. thaliana           TAGGTTGCGCTATACATATGAAAGAATATACAATAATGA
N. tabacum            TGGGTTGCGCTATATATATGAAAGAGTATACAATAATGA
N. alba               TGGGTTGCGCCATACATATGAAACAGTATACAATAATGA
A. trichopoda         TGGGTTGCGCCATACATATGAAACAGTATACAATAATGA
P. thunbergii         TGGGTTGCGTCATACATAAAGAACACTATACAATGAGAG
C. taitungensis       AGGGTTGCGCCATACATAAAGAACATTATACAATAATAG
W. mirabilis          TGGGTTGCATTATATGGAAAAAACAATCTAAAATGATAG
A. evecta             TGGGTTGCATTATACAGAAAATAATTTATAGAATACTAG
A. capillus-veneris   TTAGTTGCACCCCGCATCGGACGCGGTATAAAATAATAA
P. nudum              TGGGTTGCATCATATAGCAACTGCAATATAAAATAATAG
H. lucidula           TGGGTTGCATCACGTATCAAAAGCAATATACAATGATAA
P. patens             TGAGTTGCATCAAATGTAGAAAATAATATACAATAATAC
M. polymorpha         TAGGTTGCATTACATATAAAAAACAATATACAATAATAA
C. vulgaris           TTAGTTGCGTCATCTATTCAAGAATGTGTATAATACAAT
                             -35                      -10
                           TTGCGC                   TACAAT
```
